# Mr. Liston on Encysted Hydrocele

**Published:** 1844-02-10

**Authors:** 


### RECORD OF MEDICAL SCIENCE.

### MR. LISTON ON ENCYSTED HYDROCELE.

Mr. Liston is inclined to believe that some of the collections of fluid in the scrotum are more intimately connected with the testicle than has generally been supposed. He observes, "Some nine or ten months since, I was consulted by a healthy-looking gentleman, beyond the middle period of life, on account of tumour of the scrotum. There was plainly fluid on both sides. The largest cyst was punctured, and gave exit to some eight or ten ounces of thin fluid, which might be compared to distilled water with a little soap diffused through it. The other side of the scrotum was punctured a few months afterwards, and, as far as I can recollect, ordinary looking serum, to the extent of five or six ounces, was discharged.

"A short time since, the patient returned, to have the original cyst again emptied. About the same quantity of fluid was drawn off, and of the same quality as at first. This fluid was examined chemically, and scarcely a trace of albumen could be detected.

"On the second day, a minute quantity was put in the field of the compound microscope, and my surprise was great indeed when it appeared quite full of spermatozoa; there were, besides, to be detected some of the primitive cells, in which the spermatozoa are developed, and a certain number of mucous globules.

"It is to be regretted that the microscopic examination did not take place immediately after the fluid was obtained, so as to have ascertained whether the animalcules presented their usual liveliness of motion."

In a postcript, Mr. Liston informs us that the preceding observation has been confirmed by the examination of the fluid from a small cyst above the testicle of a man 33 years of age. The fluid here was nearly colourless, and contained numerous spermatozoa, some of which continued to move for a considerable time after the cyst was evacuated.

